# Editorial: Molecular Mechanisms of Bacterial Clinical Pathogens Tolerance and Persistence Under Stress Conditions: Tolerant and Persister Cells

**DOI:** 10.3389/fmicb.2021.705092

**Published:** 2021-07-26

**Authors:** Rodolfo García-Contreras, María Tomás

**Affiliations:** ^1^Department of Microbiology and Parasitology, Faculty of Medicine, National Autonomous University of Mexico, Mexico City, Mexico; ^2^Microbiology Department-Research Institute Biomedical a Coruña (INIBIC), Hospital a Coruña (CHUAC), University of a Coruña, A Coruña, Spain

**Keywords:** persistence, tolerance, stress, pathogenic bacteria, infections

Although resistance of bacteria against antibiotics represents the main focus of research for finding new and effective antibacterial therapies to combat recalcitrant infections (López-Jácome et al., 2019), tolerance and persistence are also relevant, since they promote antibiotic failure and the establishment of chronic infections. Tolerance is the intrinsic ability of bacteria to withstand high concentrations of antimicrobials to which they have not been previously exposed, and persister cells are a subset of bacterial populations that are temporarily dormant and hence impervious to the action of antibiotics without having genetic changes and without being resistant (Wood, 2017).

This Research Topic deals with antibiotic tolerance and persistence and its relationship to stress in pathogenic bacteria. It consists of eight works. In the first one it was demonstrated that exposure to quaternary ammonium induces the formation of persisters in *Streptococcus mutans*. Furthermore, the authors characterized the formation of those persisters in biofilms as well as the phase of reconversion of the persisters into regular replicating cells. Their findings revealed that persister resuscitation involves a long lag phase, metabolic activation and an exacerbated production of glucans. Those processes depend on quorum sensing (QS) and the VicRK, a two-component system which is responsible for the activation of genes encoding the biosynthetic pathway for exopolysaccharides. This work suggests that inhibiting QS and VicRK could be a way to decrease the rate of persister resumption and therefore they could become novel targets for anti-persister therapy against this important periodontal pathogen Lu et al.

In another work from this Research Topic Yan et al. in the role of the O-antigen in growth, stress resistance, and virulence for *Actinobacillus pleuropneumoniae* was studied. This research showed that the two-component system CpxAR promotes the transcription of *wecA*, by the binding of CpxR to its promoter, this gene encodes an enzyme that participates in O-antigen repeating unit biosynthesis. The importance of the O-antigen in growth, stress resistance, and virulence was evidenced by using *cpxAR* and *wecA* mutants, which were more sensitive to serum, oxidative and osmotic stresses than the wild-type and complemented strains and also less virulent in mice, reaching lower loads in their lungs.

One of the main bacterial pathogens responsible for chronic long-term infections and whose eradication requires prolonged treatments with several antibiotics is *Mycobacterium tuberculosis*. In their work, Sebastian et al. investigated the physiological changes of the surviving bacterium upon the prolonged exposure to rifampicin, they showed that the remaining cells developed a thickened capsular outer layer (TCOL) that was hydrophilic, highly negatively charged, and rich in polysaccharides. TCOL reduced the permeability and uptake of rifampicin. Whether a similar adaptation of *M. tuberculosis* exposed to rifampicin during lung infections in patients occurs remains to be determined.

One key aspect that contributes to the establishment of chronical *M. tuberculosis* infections is its ability to generate persister cells, in this regard the expression of toxins from toxin antitoxin modules is able to arrest growth in a bacteriostatic way, and the genome of pathogenic bacteria and in particular of Mycobacteria contain several of these modules (Fernández-García et al., 2016). In their work, Sharma et al. performed a detailed characterization of the toxin VapC21, finding that in addition to its interaction with its cognate antitoxin VapB21, it can also be neutralized by VapB32. They showed that the deletion of the toxin gene has not got important effects on either *in vitro* or *in vivo* growth in mice infections and in the tolerance to diverse stressors. In contrast, its overexpression promotes the generation of persister cells against amikacin, streptomycin, and ethambutol.

Another piece of the topic is a review about the importance of tolerance and persistence to drugs in the fight against *M. tuberculosis* infections Boldrin et al. This review compiles the information regarding the action mechanisms underlying those phenomena, including its intrinsic asymmetric cell division, that causes heterogeneity in size, composition, and properties, including tolerance against antibiotics. The importance of the alternate sigma factors SigE for the promotion of bacterial dormancy is considered. The roles of stringent response, toxin antitoxin modules, ribosome hibernation, phase variation of genes such as *glpK*, etc, and, in addition, the roles of oxidative stress, macrophages, and metabolic processes such as triacylglycerol accumulation upon stress were also discussed.

Formation of persister cells enhances the pathogenicity of the ESKAPE pathogens, including *Staphylococcus aureus*. In their work, Baldry et al. explored the role of phenol soluble modulins (PSM), four small amphipathic peptides with surfactant properties, alone and in combination in the persister formation for ciprofloxacin in *S. aureus*. Consistent with previous works (Xu et al., 2017; Bojer et al., 2018), modulines decreased persister formation in planktonic cells and in biofilms, with PSMα2 and the combination of the four modulins being the most effective. Moreover, PSM form amyloid-like fibers that are likely related to biofilm activity, and in this work the role of those fibers in modulating persister formation was ruled out.

Also in this topic, two minireview articles were included, the first one Podlesek and Bertok highlights the importance of the SOS system in the generation of persisters and tolerance in four of bacterial pathogens. The SOS response is a DNA repair pathway that is inducible by DNA damaging agents, and that is controlled by the repressor LexA and by the inducer RecA, The authors indicate that the only known direct link between the SOS response and persistence is the repression of the expression of the toxin gene *tisB* that forms the TisB/IstR toxin antitoxin module by LexA, a# repression that stops upon DNA damage, leading to an elevation of TisB levels that exceed IstR promoting dormancy and hence persistence.

The second minireview also deals with the importance persistence and tolerance against antibiotics in bacterial biofilms, focusing on *P. aeruginosa* Soares et al. First, it was discussed that the deepest biofilm layers are less exposed to nutrients and oxygen layers and trigger the generation of persisters, through the activation of mechanisms including the stringent and the SOS responses, toxin-antitoxin modules, and QS. Those persister cells are the main factor producing antibiotic failure. Then they discuss the current research about the effects of antibiotics in killing biofilm cells and eradicating persisters. They point out that very diverse models of biofilms and experimental conditions had been used, producing results that are difficult to compare and to extrapolate to the biofilm formed *in vivo*. The tolerance against different kinds of antibiotics in biofilm cells involves different morphological and physiological changes and varies depending on the antibiotics, with meropenem and colistin being more robust; furthermore, the combination of colistin and other antibiotics such as ciprofloxacin and meropenem is effective against biofilms and the persisters. Finally, the translation of the relevant experimental findings to the clinic is encouraged.

## Author Contributions

MT and RG-C wrote the manuscript. Both authors contributed to the article and approved the submitted version.

## Conflict of Interest

The authors declare that the research was conducted in the absence of any commercial or financial relationships that could be construed as a potential conflict of interest.

## Publisher's Note

All claims expressed in this article are solely those of the authors and do not necessarily represent those of their affiliated organizations, or those of the publisher, the editors and the reviewers. Any product that may be evaluated in this article, or claim that may be made by its manufacturer, is not guaranteed or endorsed by the publisher.
